# Assessment of risk priorities by cause of construction safety accidents: A case study of falling accidents in South Korea

**DOI:** 10.1016/j.heliyon.2024.e40303

**Published:** 2024-11-08

**Authors:** Seunghyun Son, Youngju Na, Bumjin Han

**Affiliations:** a Mokpo National University, Republic of Korea; b U1 University, Republic of Korea; c Dong Seoul University, Republic of Korea

**Keywords:** Risk assessment, Safety management, Fall accident, Preventive measures, Construction safety

## Abstract

In the construction industry, despite the development of technology and the efforts of companies, safety accidents are frequent, and the types of accidents are also diversified. In particular, when looking at the accident rates of the construction industry, the number of deaths from fall accidents accounts for a very high proportion. To resolve this, various measures to prevent fall, such as installation of safety railings and safety nets, have been proposed at the national level, but the effect is very insignificant. Therefore, it is necessary to establish measures for safety management and to propose prevention techniques by in-depth analysis of the causes of fall accidents through actual accident cases at the construction sites. The purpose of this study is to assess the risk of the cause of fall accidents for sustainable safety management at construction sites. To this end, data collection of fall accident cases at domestic construction sites, risk assessment by cause, and fall accident prevention techniques are conducted in order. This study was conducted on fall accident cases that occurred at a height of more than 2m. The results of this study will contribute to substantially reducing fall accidents at construction sites in South Korea. Additionally, it is used as basic data for improving Korea's construction safety management system.

## 1 Introduction

In recent years, as the construction industry has gradually increased in size, working methods have become more complex and safety management systems have diversified [[1], [2], [3]]. In particular, since construction work has to be completed within the required time, there is a tendency for the construction to proceed unreasonably when there is insufficient time [[4], [5], [6]]. In the case of urban constructions, risk factors are becoming more diverse due to continuously increasing external variables and constraints [7,8]. As a result, the risk of construction safety accidents is increasing [4,5,[7], [8], [9]].

Currently, the construction industry is known as a disaster-prone industry that accounts for more than one-third of all industrial accidents in Korea [10], and prevention of construction accidents is recognized as a national level task [11]. In the past 10 years, the accident rate has been decreasing in most industries, whereas it has been increasing in the construction industry [12].

According to data from the Korea Occupational Safety and Health Agency, the mortality rate of all industries decreased from 7.05 % in 2009 to 4.84 % in 2017, whereas it increased from 6.55 % in 2009 to 8.42 % in 2017 for the construction industry. It appears to have increased by about 1.87 % [13]. Currently, the number of deaths from fall accidents accounts for a very high proportion in the construction industry [[14], [15], [16], [17]].

For example, in 2018, out of the total number of injuries in the construction industry (26,486), the number of injuries due to fall accident was 9191 (34.70 %), accounting for the highest proportion [13].

**Fig. 1:**
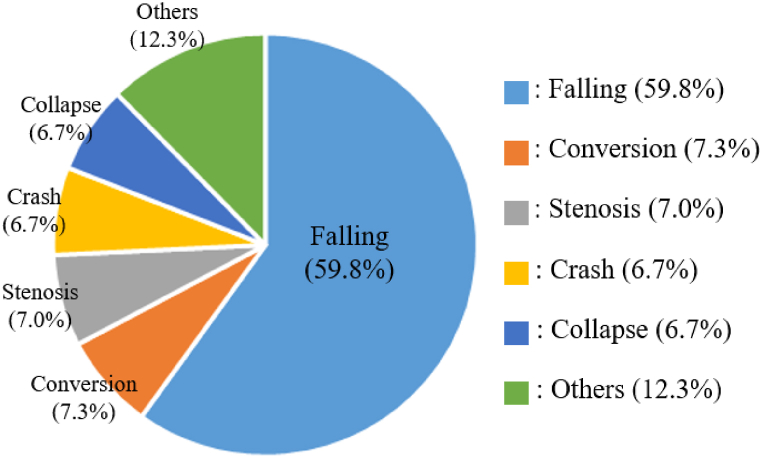
Deaths by cause of disasters at construction sites in 2022 [18].

As shown in Fig. 1, according to the current status of deaths in the construction industry by the Ministry of Employment and Labor in Korea announced in January 2023, falling accounted for 59.8 % of all accident types [18].

As such, most deaths in the construction industry occur in fall accidents. For effective safety management at construction sites, it is necessary to analyze the in depth causes of the actual fall accident cases, establish measures to prevent this, and propose prevention techniques. The data collected in this study are cases of fall accidents that have been compensated for in Korea's industrial accident insurance.

Through insurance payment information, the collected data can confirm the actual number of days due to medical care for the injured person and number of days lost due to physical disability grade. The frequency and severity of a fall accident are calculated using the collected data. Through this, it is possible to evaluate the risk of a fall accident. Therefore, the purpose of this study is to assess the risk of the cause of fall accidents for sustainable safety management at construction sites.

## 2 Methodology

This study evaluates the risk of each cause and proposes preventive measures for the case of fall accidents at construction sites. First, the research on the causes and risk assessment of fall accidents of the construction industry are reviewed. Through this, the main factors of fall accidents and the risk assessment method to be applied to this study are determined.

**Fig. 2:**
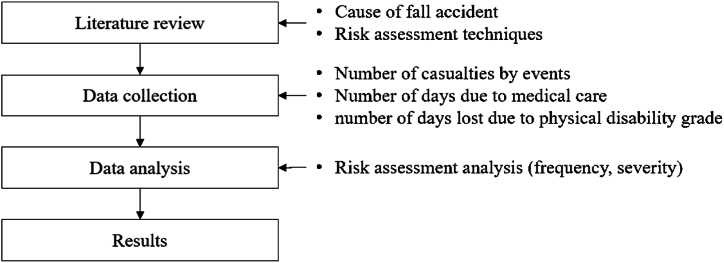
Methodology.

Second, the fall disaster field data is collected from domestic construction sites. At this time, the number of casualties by cases, number of days due to medical care, and number of days lost due to physical disability grade are collected. Third, a risk assessment is performed based on the collected data. At this time, the risk assessment for each cause is carried out by comprehensively considering the frequency and depth of occurrence. Through this, when establishing a fall disaster preventive measure, a high-risk unit case is identified, and a risk priority number (*RPN*) is derived. Fourth, using the analyzed results, a preventive measure and safety management technique regarding sustainable safety management at construction sites is proposed. Fig. 2 shows the method and procedure of this study.

## 3 Literature review

### 3.1 Cause of fall accident

When a fall accident occurs, laborers mostly end up with a fatal injury or death [14,19,20]. Cattledge et al. [14], Chi and Wu [15], Chi and Chen [16], and Janicak [17] reported that among the type of accidents occurring at construction sites, accidents caused by falls are the most common and cause the most fatal injuries. In order to reduce such fall accidents at construction sites, a more in-depth analysis of the cause of accidents is required. As a result of analyzing data on fall accidents at construction sites, Bobick [21] concluded that working on roofs, scaffolds, and openings caused the most fatal injuries.

Dodge [22] reported that when working at high places, such as scaffolds, openings, and work footings, fall accidents occurred more frequently as it is more difficult to communicate between la-borers of various nationalities. Huang and Hinze [23] argued that the work environment such as conditions of working surfaces, including scaffolds, ladder, roof and openings, and working heights are most closely related to fall accidents. Yim et al. [24] argued that most of the fall accidents occur at the scaffold. They developed a rotary safety ring to prevent a fall accident on the scaffold.

In addition, Shin et al. [25] analyzed the causes of death by type of occurrence in the construction industry for five years from 2009 to 2013, and stated that fall accidents occur due to slipping, tripping, miss-stepping, and imbalance on ladders; scaffolds; stairs; openings; and roofs. As such, most of the studies attempted to analyze the cause of the accident by classifying the cases of the fall accidents [[21], [22], [23],^25^].

With reference to aforementioned studies, in this study, the data of construction site fall accidents are classified into five cases (ladder, scaffold, work footing, opening, and roof), and the frequency and severity are comprehensively considered. Through this, the study aims to identify the high-risk unit case of fall accidents and derive the *RPN* for sustainable safety management at construction sites.

### 3.2 Risk assessment techniques

In general, risk assessment involves identifying hazard or risk factors in advance (identification), estimating the risk, and taking measures according to the magnitude of the estimated risk [26]. In the Guidelines for the implementation of safety management work for construction works of the Ministry of Land, Infrastructure and Transport in the Republic of Korea, the frequency and severity of accidents are defined as risk [27].

Risk assessment is conducted in various forms depending on the evaluation objective, goal, or method of evaluation [28]. In this regard, several references are reviewed, and risk assessment techniques are divided into qualitative and quantitative methods as shown in Table 1 [[28], [29], [30], [31], [32]].

**Table 1:** Risk assessment technique.

Assessment	Technique
Qualitative	Checklist
	What-if
	Preliminary hazard analysis
Quantitative	Failure mode and effect analysis
	Event tree analysis
	Fault tree analysis

Qualitative methods in Table 1 include Checklist, What-if, and Preliminary Hazard Analysis (PHA) [[28], [29], [30]]. In general, these qualitative methods enumerate risk factors and evaluate the risk by discussing the frequency and severity of the accidents [28,29].

These evaluation methods are useful as a method of identifying risk factors or analyzing the cause of an accident, but it has a disadvantage that it can omit complicated or difficult to predict items [30]. Quantitative methods in Table 1 include failure mode and effect analysis, event tree analysis, and fault tree analysis (FTA) [[30], [31], [32]]. These quantitative methods evaluate the cause or risk of an accident by quantitatively calculating the frequency and depth caused by the risk factors [31,32].

In addition, the Risk Assessment Matrix (RAM) is a tool used to evaluate risks and determine priorities by grading severity and likelihood of occurrence. It facilitates efficient decision-making across various industries and organizations by systematically assessing and prioritizing risks [33].

In this way, various risk assessment techniques evaluate risk by analyzing the frequency and depth of accidents of commonly collected data. Therefore, in this study, for sustainable safety management at construction sites, *RPN* is derived by quantitatively calculating the frequency and depth of accidents caused by cases based on statistical data of fall accidents for 10 years (2010–2019) that was collected by industrial accident compensation insurance of Occupational Safety and Health Agency in Republic of Korea, and by multiplying the two factors.

## 4 Data collection

In this study, statistical data of industrial accident compensation insurance due to fall accidents from 2010 to 2019 of Occupational Safety and Health Agency in Republic of Korea was collected. Among them, data of 2816 casualties (including the deceased), corresponding to the five main cases in the fall accident were selected. The demographics of the collected samples are shown in Table 2. In the samples shown in Table 2, over the past 10 years, 2594 men (92.10 %) and 222 women (7.90 %) were covered by the industrial accident compensation insurance due to fall accidents. At this time, the number of days due to medical care, physical disability grade, and accident occurrence type were collected for each injured person.

**Table 2:** Demographics.

Variable	Category	N	%
Gender	Male	2594	92.10
	Female	222	7.90
Age	≤24 years	318	11.30
	25–34 years	625	22.20
	35–44 years	819	29.10
	45–54 years	527	18.70
	≥55 years	513	18.20
	Unknown	14	0.50
Career	≤5 years	316	11.22
	6–9 years	1090	38.71
	10–14 years	1032	36.65
	≥15 years	378	13.42
Company size	≤5 individuals	1118	39.70
	6–9 individuals	485	17.22
	10–29 individuals	658	23.37
	30–49 individuals	220	7.81
	50–99 individuals	184	6.53
	100–499 individuals	132	4.69
	500–999 individuals	15	0.53
	≥1000 individuals	4	0.14

**Fig. 3:**
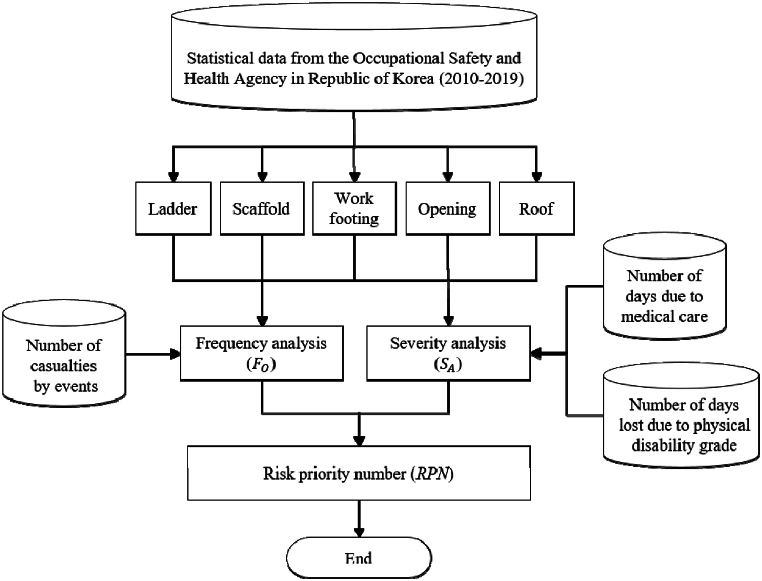
Data analysis procedure.

Based on these data, the data analysis procedure of this study is shown in Fig. 3. As shown in Fig. 3, fall accident data from the Occupational Safety and Health Agency in Republic of Korea is classified by accident events. Frequency analysis is performed using the number of casualties by events. Severity analysis is performed using data on the number of days due to medical care for each injured person and the number of days lost due to physical disability grade. Finally, high risk events of fall accidents are identified and a risk priority number is derived.

## 5 Data analysis

### 5.1 Frequency analysis

In this study, the frequency of accident occurrence ($F_{O}$) is calculated as shown in Equation (1) to evaluate the risk of fall accidents classified by different cases. The frequency of accident occurrence ($F_{O}$) refers to the ratio of the number of casualties due to different cases ($N_{E}$) divided by the number of total casualties ($N_{T}$), as shown in Equation (1). Based on the number of total casualties, it is possible to predict the frequency of accidents due to each case.

(1) $$F_{O}=\frac{N_{E}}{N_{T}}\times 100$$

From the equation, $F_{O}$: frequency of accident occurrence, $N_{E}$: number of casualties due to different cases, $N_{T}$: number of total casualties.

Table 3 shows the frequency of accident occurrence of the collected data using Equation (1). As shown in Table 3, the frequency of accident occurrence ($F_{O}$) of each case of fall accident was ladder, scaffold, work footing, opening, and roof.

**Table 3:** Frequency distribution of accident cases.

Factor		Frequency (individuals)	$F_{O}$ (%)	Priority
Accident case	Fall from ladder	1127	40.02	1
	Fall from scaffold	723	25.67	2
	Fall from work footing	540	19.18	3
	Fall from opening	236	8.38	4
	Fall from roof	190	6.75	5
Total		2816	100	

In particular, out of the 2816 total number of casualties, the number of casualties due to the case of ladder occupied the highest ratio with 1127 (40.02 %), and the number of casualties due to the case of fall accident from roof was 190 (6.75 %), accounting for the smallest percentage.

### 5.2 Severity analysis

In this study, severity ($S_{A}$) is calculated as shown in Equation (2) for the risk assessment of fall accidents for each case. $S_{A}$ is calculated by dividing disaster intensity index (DI) by $N_{E}$. This value is calculated based on the actual number of days due to medical care and the number of days lost due to the physical disability grade. It is possible to predict the severity of accidents due to each case [34].

(2) $$S_{A}=\frac{\sum DI}{N_{E}}=\frac{\sum convert\left(D_{C}+D_{L}\right)}{N_{E}}$$

From the equation, $S_{A}$: severity of fall accident, DI: disaster intensity index, $D_{C}$: number of days due to medical care, $D_{L}$: number of days lost due to physical disability grade.

At this time, in calculating the severity of the casualties, the number of days due to medical care can be distributed from 4 to 7500 days (death) [35]. According to Article 80 of the Labor Standards Act in Korea, when a casualty occurs, the number of days of due to medical care is 7500 [[34], [35], [36]]. This can result in a very high *RPN* regardless of the frequency of accident occurrence for different cases. Therefore, severity of the accident is calculated using the DI of 10 levels as shown in Table 4.

**Table 4:** Calculation method of DI.

DI	Days lost	Basis of calculation
1	4∼10	sum of the number of days due to medical care and number of days lost due to physical disability grade
2	11∼30	
3	31∼90	
4	91∼180	
5	181∼360	
6	less than 1000	
7	less than 3000	
8	less than 7500	
9	7500	physical disability 1–3 grade
10	7500	death

The DI is divided into 10 levels as shown in Table 4, which is calculated from sum of the number of days due to medical care ($D_{C}$) and the number of days lost due to physical disability grade ($D_{L}$) according to the 14 physical disability grades from the Labor Standards Act [34]. $S_{A}$ of each case using DI is shown in Table 5.

**Table 5:** Severity of accident cases.

Factor		$Ave\left(D_{C}\right)$	$Ave\left(D_{L}\right)$	∑DI	$S_{A}$	Priority
Accident case	Fall from roof	1814	4500	707	3.721	1
	Fall from opening	2249	3000	861	3.648	2
	Fall from ladder	10,684	5250	4020	3.567	3
	Fall from scaffold	6750	4500	2574	3.560	4
	Fall from work footing	5100	1500	1900	3.519	5

As shown in Table 5, $S_{A}$ was in the order of roof, opening, ladder, scaffold, and work footing. The severity of fall accidents from roof was the highest (3.721), and from work footings was the lowest (3.519).

To explain this in more detail, in the case of the roof, 12 individuals (6.3 %) of the 190 fall casualties were with physical disability grades of 1–3 or higher (including death), and it was very high compared to other cases. Accordingly, the number of days lost was calculated high. In other words, falls do not occur often on roofs, but when a fall occurs, workers are seriously injured.

In contrast, in the case of work footing, out of 540 fall casualties, 3 individuals (0.6 %) were with physical disability grades of 1–3 or higher (including death), and it was lower than that of the other cases. These results indicate that falls from work footings often occur, but only minor accidents occur.

### 5.3 Risk assessment of accident cases

**Fig. 4:**
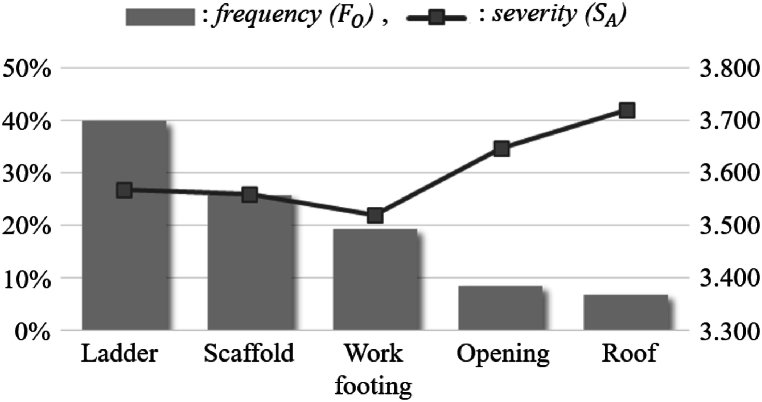
Frequency and severity of cases leading to fall accidents.

In this section, the risk for each case is evaluated based on the previously analyzed results. Through this, high-risk unit cases of fall accidents are identified, and *RPN* is derived. In this study, Equation (3) is used to evaluate the risk of fall accidents due to each case. As shown in Equation (3), the risk of fall accident ($R_{A}$) can be calculated by multiplying $F_{O}$ and $S_{A}$. Fig. 4 and Table 6 are the results of the risk assessment due to each case of fall accidents.

(3) $$R_{A}=F_{O}\times S_{A}$$

In this equation, $R_{A}$: risk of fall accident, $F_{O}$: frequency of accident occurrence, $S_{A}$: severity of fall accident.

**Table 6:** *RPN* by accident cases.

Factor		$F_{O}$	$S_{A}$	$R_{A}$	Priority
Accident case	Fall from ladder	40.02	3.567	1.428	1
	Fall from scaffold	25.67	3.560	0.914	2
	Fall from work footing	19.18	3.519	0.675	3
	Fall from opening	8.38	3.648	0.306	4
	Fall from roof	6.75	3.721	0.251	5

As shown in Fig. 4 and Table 6, the risk of fall accident ($R_{A}$) was in the order of ladder, scaffold, work footing, opening, and roof. The unit event attributable to the highest risk of accident is ladder (1.428), which should be considered foremost when establishing fall accident preventive measures. In addition, thorough management is required when a worker is seriously injured, such as a fall from an opening and a roof. As such, it is necessary to consider both frequency and depth in order to reduce fall accidents occurring in construction sites, and in particular, it is considered that special management of tasks directly connected to serious disasters such as death is required.

As such, in this study, the risk assessment due to each case was conducted using actual accident cases for sustainable safety management at construction sites. In particular, since this study used actual accident cases that occurred at construction sites, it is considered to be more effective than the method using the existing questionnaire survey. In the case of $F_{O}$, it can be analyzed as a quantitative number because it is defined as the ratio of the number of casualties due to each case to the number of total casualties, and it is judged to be more objective than surveys and interviews. In the case of $S_{A}$, the severity of the fall accident was defined by reflecting the number of days due to medical care of accident occurrence and the number of days lost due to physical disability grade; the severity of accidents can be identified when an accident occurs in real life.

## 6 Preventive measures for all accidents

As a result of evaluating the risk comprehensively considering the frequency and depth of the cases, shown in Table 7, the cases with high frequency of accident occurrence appear as ladder, scaffold, and work footing, respectively. Most accidents are due to negligence of the laborers, such as slipping, miss-stepping, noncompliance with work instructions, and noncompliance with safety-related laws, and they occur in various forms regardless of the type of work.

**Table 7:** The main cause of fall accidents.

Accident case	Characteristic	The main cause of the accident
Fall from ladder	High frequency of occurrence	slipping, miss-stepping, noncompliance with work instructions, noncompliance with safety-related laws
Fall from scaffold		
Fall from work footing		
Fall from opening Fall from roof	High disaster severity	defects and damages to parts of safety equipment, damages caused by deteriorated roof, damaged and loosened opening cover

In particular, many accidents occur at a height that is relatively low compared to other cases, that is, at a level that laborers themselves perceive as not dangerous. In addition, as shown in Table 7, the causes with high severity of fall accident are roof and opening. Main causes of these accidents were defects and damages to parts of safety equipment, damages caused by deteriorated roof, damaged or loosened opening covers, and lack of covers; safety railings; and caution signs. Most accidents occurred due to poor management and installation of safety equipment.

In consideration of the results of this analysis, the preventive measures for fall accidents are as follows: first, for work on ladders, scaffolds, and work footings, the safety management system should be further strengthened, such as assigning a supervisor and conducting the work under supervision to prevent fall accidents, conducting work in groups of two, and wearing personal protective equipment such as hard hats. In addition, efforts should be made to raise the level of awareness of laborers' safety through continuous safety education.

Second, for work on the roof and openings, protective measures such as installing a fall protection net and safety railings should be reinforced at locations where there is a risk of falling, and additional safety facilities must be secured such as when it is difficult to install safety rails, install a safety belt and secure safety belt hanging sites to ensure the safety of the laborers working at heights.

Third, as described in Table 6, the *RPN* was in the order of ladder, scaffold, work footing, opening, and roof. The *RPN* of fall accidents from ladder was the highest (1.428), and from roof was the lowest (0.251). For the sustainable safety management at construction sites by reducing fall accidents, it is necessary to establish fall accident prevention measures that consider both frequency and severity. In addition, tasks that are directly connected to serious disasters such as casualties require special management with priority.

## 7 Conclusion

This study was conducted for evaluating the cases leading to fall accidents for sustainable safety management at construction sites. To this end, statistical data on industrial accident compensation insurance due to fall accidents for 10 years from 2010 to 2019 of Occupational Safety and Health Agency in Republic of Korea was collected. The collected data were used to quantitatively analyze the frequency and depth of fall accidents, and the risk of fall accidents was assessed by comprehensively considering these data. The results of this study are as follows:

First, $F_{O}$ of fall accidents due to each case was in the order of ladder, scaffold, work footing, opening, and roof. Fall accidents on the ladder were the highest at 40.02 %. This result suggests that many accidents occur at relatively low heights, that is, at a level that laborers themselves perceive as not dangerous. In the case of ladders, scaffolds, and work footings with high occurrence, efforts to reduce the fall accidents must be made, including continuous safety education to raise safety awareness, thorough supervision of laborers negligence, work in groups of two, and wearing personal protective equipment.

Second, $S_{A}$ was in the order of roof, opening, ladder, scaffold, and work footing. Fall accidents do not frequently occur when working at a relatively high position; however, it has been suggested that fall accident occurs, it directly leads to serious disasters, such as casualties. In the case of roof (3.721) and opening (3.648) with high severity of fall accidents, safety facilities, such as fall protection nets and safety railings, should be installed at high risk locations, and on-site safety managers should conduct real time monitoring and control of the facilities for immediate repair of deteriorated safety facilities.

Third, considering the frequency and severity comprehensively, the *RPN* is ladder, scaffold, work footing, opening, and roof. The *RPN* of fall accidents from ladder was the highest at 1.428, and from roof was the lowest at 0.251. On site safety managers should consider the risk priority when establishing a safety management plan, and put extra efforts in an in depth management for roofs and openings that are directly linked to serious disasters such as casualties.

The results of this study will be used as a basic data for improving the safety management system at construction sites. In the future, it will contribute to effective and sustainable safety management at construction sites.

## CRediT authorship contribution statement

**Seunghyun Son:** Writing – original draft, Visualization, Validation, Software, Methodology, Formal analysis. **Youngju Na:** Visualization, Resources, Investigation, Formal analysis, Data curation. **Bumjin Han:** Writing – review & editing, Supervision, Methodology, Formal analysis, Conceptualization.

## Data availability statement

Authors confirm that all relevant data are included in this manuscript, and all sources are well cited.

## Funding

This research was supported by a grant (NRF-2021R1C1C2091677) from the 10.13039/501100003725National Research Foundation of Korea by 10.13039/501100003621Ministry of Science, ICT and Future Planning.

## Declaration of competing interest

The authors declare that they have no known competing financial interests or personal relationships that could have appeared to influence the work reported in this paper.
